# Research Progress on Gene Editing Based on Nano-Drug Delivery Vectors for Tumor Therapy

**DOI:** 10.3389/fbioe.2022.873369

**Published:** 2022-03-28

**Authors:** Shiwen Xi, Yong-Guang Yang, Jian Suo, Tianmeng Sun

**Affiliations:** ^1^ Key Laboratory of Organ Regeneration and Transplantation of Ministry of Education, Institute of Immunology, The First Hospital, Jilin University, Changchun, China; ^2^ Gastrointestinal Surgical Department, The First Hospital, Jilin University, Changchun, China; ^3^ National-local Joint Engineering Laboratory of Animal Models for Human Diseases, Changchun, China; ^4^ International Center of Future Science, Jilin University, Changchun, China

**Keywords:** gene editing, tumor, nano-drug delivery vectors, gene therapy, gene-editing technique

## Abstract

Malignant tumors pose a serious threat to human health and have high fatality rates. Conventional clinical anti-tumor treatment is mainly based on traditional surgery, chemotherapy, radiotherapy, and interventional therapy, and even though these treatment methods are constantly updated, a satisfactory efficacy is yet to be obtained. Therefore, research on novel cancer treatments is being actively pursued. We review the classification of gene therapies of malignant tumors and their advantages, as well as the development of gene editing techniques. We further reveal the nano-drug delivery carrier effect in improving the efficiency of gene editing. Finally, we summarize the progress in recent years of gene editing techniques based on nano-drug delivery carriers in the treatment of various malignant tumors, and analyze the prospects of the technique and its restricting factors.

## Introduction

Malignant tumors have specific biological characteristics, including abnormal cell differentiation and proliferation, uncontrolled growth, invasion, and metastasis. Due to their different genotypic changes such as EGFR mutation and ALK gene mutation, different tissues and organs involved, and various stages, the tumors’ responses to various treatments differ accordingly. Traditional treatments include surgery, chemotherapy, radiotherapy, and interventional therapy. Almost all traditional treatment methods face difficulty to completely eradicate the tumor, as their efficacy reaches a plateau that is difficult to break through. Furthermore, the toxicity and side effects caused by traditional radiotherapy and chemotherapy—such as digestive tract reaction, blood system changes, and bone marrow suppression—also severely restrict their wide clinical application. Hence, the search for novel cancer treatments continues.

## Classification of Gene Therapies for Malignant Tumors and its Advantages as a Novel Approach to Treat Tumors

Gene therapy refers to the introduction of exogenous normal genes to correct or compensate diseases caused by gene defects and abnormal genes, to achieve the purpose of treatment. Gene therapy for malignant tumors includes suicide gene therapy, corrective gene therapy, and toxin/apoptosis-induced gene therapy (Karjoo et al., 2016). The thymine kinase (TK) gene commonly used today is based on this principle. Corrective gene therapy is a method applying therapeutic agents, such as siRNA or, miRNA, or gene-editing tools, into tumor cells to change their gene expression and inhibit their proliferation (Senzer et al., 2013; Tabernero et al., 2013; Chen et al., 2015; Liu et al., 2015). Toxin/apoptosis-induced gene therapy is a more direct approach to inducing tumor cell death through the delivery of genes producing toxic substances, such as TNF-α.

Study has shown that the malignant tumor is a fatal disease that involves multiple genes and changes the epigenetics of the entire genome (Garraway and Lander, 2013). These gene mutations usually promote the occurrence and development of tumors (Sánchez-Rivera and Jacks, 2015). In traditional lesion resection, chemotherapy, and radiation therapy, the treatment effects are limited, and side effects to the patients’ body are significant. Gene therapy, as a novel treatment, has the advantages of high specificity and targeting, leading to less side effects. It is capable of entirely eliminating the tumor, and has therefore attracted the attention of scientists and clinicians. In the past 2 decades, with the help of high-throughput sequencing technology, numerous genes related to the occurrence and development of malignant tumors have been identified (Pon and Marra, 2015). Based on these advances, gene therapy has brought great hope for the treatment of malignant tumors by adjusting gene expression and correcting mutations. More than 2,000 clinical trials have been conducted so far, two-thirds of which were for the treatment of malignant tumors (Bertrand et al., 2014), and many of which yielded encouraging results.

## Occurrence and Development of Gene-Editing Technology in Gene Therapy

Gene editing is a technique or process that enables precise modification of a specific target gene in an organism’s genome. In the early stage, genetic engineering technology was relatively crude, and only exogenous or endogenous genetic material could be randomly inserted into the host genome. With technological progress, gene editing became more sophisticated, allowing the desired gene to be edited at a specific point. The technology relies on the engineered nuclease, also known as “god’s knife,” at a specific location in the genome to produce a site-specific double-stranded break (DSB), induction of organisms through the homologous end connection, or homologous recombination to repair the DSB, the error-prone repair process, leading to a targeted mutation. This targeted mutation is referred to as gene editing, and has shown great potential in gene research, gene therapy, and genetic improvement owing to its high efficiency in site-directed genome editing.

Homologous recombination was the first technique used to edit the cell genome. It is the exchange (recombination) of genetic information between two similar (homologous) strands of DNA. It involves producing and isolating fragments of DNA with sequences similar to those of the parts of the genome to be edited, injecting these fragments into monocytes, or making them be absorbed by the cell with special chemicals. These fragments, once inside the cell, can be recombined with the cell’s DNA to replace the targeted parts of the genome. The disadvantage of this method is its high error rate and low efficiency.

To overcome this problem and create site-specific double strand breaks, four different types of nucleases have been bioengineered. They are giant nucleases (meganuclease), zinc finger nucleases (ZFNs), transcriptional activating-like effecting-factor nucleases (TALEN), and clustered regularly spaced short palindromic repeats associated systems (CRISPR/Cas). CRISPR-Cas is a prokaryotic immune system that endows prokaryotes with resistance to foreign genetic material, such as those present in plasmids and phages. It is hence an acquired immune system. According to different Cas proteins, the CRISPR/Cas system can be divided into type i, ii, and iii. Cas9 belongs to the type ii CRISPR system and is the currently most widely used gene-editing tool. The Cas9 nuclease consists of two conserved nuclease domains, HNH and RuvC. Under the guidance of crRNA and tracrRNA, specific cleavage of DNA double strands can be performed. The cleavage sites are usually located 3 nt upstream of the protospacer-adjacent motif (PAM) (Sapranauskas et al., 2011). Researchers fused crRNA and tracrRNA to create chimeric single guide RNA (sgRNA) (Jinek et al., 2012). Under the guidance of sgRNA, Cas9 can be directed to a target near the PAM sequence to form DSB at a specific site. Host cells respond to double strand breaks through two different mechanisms, non-homologous end joining (NHEJ) and homology-directed repair (HDR), leading to insertion/deletion and frameshift mutations in the target DNA. When donor DNA is provided as a homologous recombination template, cells repair in the manner of HDR, which can achieve precise insertion, deletion, or replacement of bases at specific sites (Gasiunas et al., 2012). Emmanuelle Charpentier and Jennifer Doudna, who developed the CRISPR/Cas9 gene-editing technology, won the 2020 Nobel Prize in Chemistry.

Currently, gene-editing technology is widely employed in biological and medical research. Because malignant tumors are caused by genomic changes of tumor cells, gene-editing technology can be used in the research field of malignant tumors to explore the potential mechanism of their occurrence and development. In recent years, gene-editing technology based on the CRISPR/Cas9 system has also been applied in clinical trials of a variety of malignant tumors, showing significant potential (Table 1). The CRISPR/Cas9 system can be introduced in three typical forms: plasmid DNA (pDNA), mRNA, and ribonucleoprotein (RNP, a complex of cas9 protein with sgRNA) (Yang et al., 2020; Duan et al., 2021). The pDNA-based CRISPR/Cas9 system generally performed by integrating the both cas9 protein and sgRNA encoding plasmids into a single vector, to avoid multiple transfections. However, the gene fragment size encoding the CRISPR/Cas9 system and the pDNA are often too large (∼4.3 and ∼10 kbp), resulting in a low transfection efficiency. Cas9 mRNA, which can be prepared by *in vitro* transcription, is another possibility for delivered cargo. The Cas9 mRNA directly translated into protein in the cytoplasm to exert their genome editing function after being transferred into the cells. However, the low stability and limited expression time of mRNA are the main limitation for its application. The RNP-based CRISPR/Cas9 system is considered as the most straightforward strategy, which can quickly start the genome editing without the process of transcription and/or translation following being transferred into cells. However, the activity and the intracellular delivery efficiency of the purified Cas9 protein with large molecular weight become the main challenges. (Zhang S. et al., 2021; Duan et al., 2021). Many nano-drug delivery platforms have been developed for the CRISPR/Cas9 system (Table 2).

**TABLE 1:** Summary of clinical trials using gene editing tools for malignant tumors.

Title	Characteristics	Interventions	Status	Study results
Study of CRISPR-Cas9 Mediated PD-1 and TCR Gene-knocked Out Mesothelin-directed CAR-T Cells in Patients With Mesothelin Positive Multiple Solid Tumors	Phase 1	anti-mesothelin CAR-T cells	Recruiting	No Results Available
A Study of Metastatic Gastrointestinal Cancers Treated With Tumor Infiltrating Lymphocytes in Which the Gene Encoding the Intracellular Immune Checkpoint CISH Is Inhibited Using CRISPR Genetic Engineering	Phase 1	Biological: CRISPR/Cas9	Recruiting	No Results Available
	Phase 2			
A Safety and Efficacy Study of TALEN and CRISPR/Cas9 in the Treatment of HPV-related Cervical Intraepithelial Neoplasia^#^	Phase 1	Biological: TALEN	Unknown status	No Results Available
		Biological: CRISPR/Cas9		
Study of PD-1 Gene-knocked Out Mesothelin-directed CAR-T Cells With the Conditioning of PC in Mesothelin Positive Multiple Solid Tumors	Phase 1	Biological: Mesothelin-directed CAR-T cells	Unknown status	No Results Available
PD-1 Knockout Engineered T Cells for Advanced Esophageal	Not Applicable	Biological: CRISPR/Cas9	Completed	No Results Available
Cancer	—	—	—	—
PD-1 Knockout Engineered T Cells for Metastatic Non-small Cell Lung Cancer	Phase 1	Biological: CRISPR/Cas9	Completed	Has results
Stem Cells in NF1 Patients With Tumors of the Central Nervous System	—	Biological: CRISPR/Cas9	Suspended	No Results Available
TGFβR-KO CAR-EGFR T Cells in Previously Treated Advanced EGFR-positive Solid Tumors	Phase 1	Biological: TGFβR-KO CAR-EGFR T Cells	Not yet recruiting	No Results Available
PD-1 Knockout EBV-CTLs for Advanced Stage Epstein-Barr	Phase 1	Biological: CRISPR/Cas9	Recruiting	No Results Available
Virus (EBV) Associated Malignancies	Phase 2			
NY-ESO-1-redirected CRISPR (TCRendo and PD1) Edited T	Phase 1	Biological: NY-ESO-1 redirected autologous T cells with CRISPR edited endogenous TCR and PD-1	Terminated	No Results Available
Cells (NYCE T Cells)	—	—	—	—
A Safety and Efficacy Study Evaluating CTX130 in Subjects With Relapsed or Refractory Renal Cell Carcinoma (COBALT-RCC)	Phase 1	Biological: CRISPR/Cas9	Recruiting	No Results Available
TACE Combined With PD-1 Knockout Engineered T Cell in Advanced Hepatocellular Carcinoma	Phase 1	Biological: CRISPR/Cas9	Recruiting	No Results Available
Study of Molecular-targeted Therapy Using Zinc Finger	Phase 1	Biological: ZFN-603 and ZFN-758	Unknown status	No Results Available
Nuclease in Cervical Precancerous Lesions				
Study of Targeted Therapy Using Transcription Activator-like Effector Nucleases in Cervical Precancerous Lesions	Phase 1	Biological: ZFN-27 and ZFN-512	Recruiting	No Results Available

**TABLE 2:** Summary of different non-viral NPs delivery system for CRISPR/Cas9.

Delivery System	CRISPR/Cas9 Cargo	Advantages	Disadvantages
Lipid nanoparticle	pDNA	High biocompatibility	Limited delivery efficiency
	mRNA	Minimal immunogenicity	Complex preparation process
	RNP	Relatively facilitate large-scale production	—
	—	High safety	—
	—	Integrated delivery	—
Polymer nanoparticle	pDNA	Minimal immunogenicity	Limited delivery efficiency
	mRNA	Relatively facilitate large-scale production	Variable biocompatibility and toxicity
	RNP	High safety	—
	—	Integrated delivery	—
DNA nano-structure	RNP	Controllable size and architecture	Complex preparation process
	—	—	Poor stability of DNA carrier
Inorganic nanoparticle	pDNA	High delivery efficiency	Limited delivery efficiency
	mRNA	Minimal immunogenicity	Potential toxicity *in vivo*
	RNP	Relatively facilitate large-scale production	—
	—	Integrated delivery	—
Peptide nanoparticle	pDNA	Relatively facilitate large-scale production	Limited delivery efficiency
	mRNA	Integrated delivery	*In vivo* degradation
	RNP	—	Potential immunogenicity from foreign peptide
Nanogels	pDNA	Serum tolerance	Limited delivery efficiency
	—	High safety	—
	—	High loading capacity	—
	—	Feasible of *in vivo* application	—

## Role of Nano-Drug Delivery Vectors in Improving Gene-Editing Efficiency

Similar to drugs, gene-editing tools must circulate to eventually reach their target cells. The existence of various compounds and enzymes in human blood circulation will remove the foreign substances, which is the first obstacle for gene-editing tools to perform specific functions. The blood–brain barrier, blood–thorax barrier, and other barriers composed of dense capillary endothelial cells and a basal membrane can prevent substances from passing through. These substances must furthermore pass through the cell membrane barrier before entering the nucleus (Xu et al., 2020). Moreover, different tissues have different pH values. Both of these factors have effects and even pose difficulties in the transmission of gene-editing tools. Methods of transmission include physical methods and application carriers (Yin et al., 2017). Common physical transport methods include electroporation, microinjection, osmotic cell proliferation, and iTOP induced channel ions, mechanical cell deformation, and hydraulic jet (Liu et al., 2017). Many of these methods are accompanied by collateral damage, such as cell membrane destruction, and are therefore not suitable for *in vivo* application (Yan et al., 2021). Thus, an ideal vehicle is needed.

The ideal vector for gene delivery must meet the following criteria: 1) the vector must be able to express transgenes for a duration of time, and the expression must be precisely regulated; 2) the carrier must be easy to produce at higher titers to allow small volume transfer and must be suitable for commercial production and processing; 3) it must have specificity of target cells; 4) immunity is indolent, thus allowing repeated administration; 5) the vector must have no limit on the size of the genetic material it can transfer; 6) the vector must allow site-specific integration into the chromosomes of target cells or exist in the nucleus as epistasis; 7) vectors must be capable of transfecting both mitotic and non-mitotic cells (Somia and Verma, 2000; Mehierhumbert and Guy, 2005; Al-Hendy and Salama, 2006). Commonly used vectors include viral and non-viral vectors. Viral vectors include lentiviruses, and adeno-associated viruses, which are sometimes unstable in nature and may lead to immunogenicity and insertion mutations, or even carcinogenesis (Dai et al., 2020). Nanoparticles (NPs) are ultra-small particles with a diameter below 1000 nm, composed of a variety of materials such as lipids, polymers or metals. The properties of NPs, such as their material composition, particle size, electrical potential, and surface modification can be carefully designed for the delivery of drugs, nucleic acids, and other substances.

**FIGURE 1:**
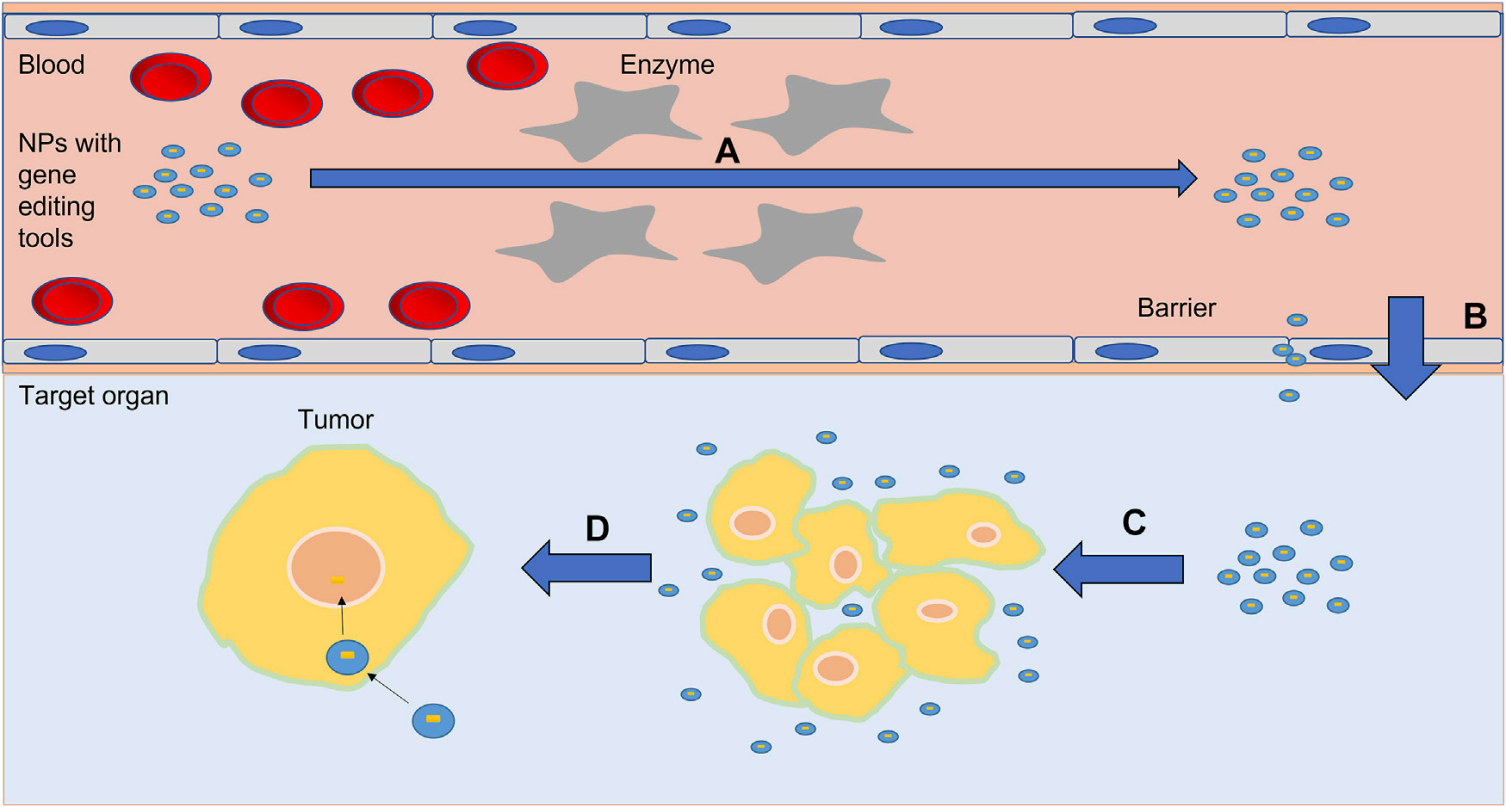
Schematic diagram of nanoparticle delivery gene editing tool into tumor cells *in vivo*. **(A)** Nanoparticles prevent gene editing tools from degradation by enzymes in circulation. **(B)** Nanoparticles cross barriers such as the blood-brain barrier with gene editing tools. **(C)** Nanoparticles accumulate around the tumor cells. **(D)** Nanoparticles enter the tumor cells and release gene editing tools.

NPs can combine nucleic acid therapy based on chemical binding or electrostatic interaction to overcome the treatment challenges of malignant tumors and other diseases (Milling et al., 2017; Riley et al., 2019). First, NPs provide protection against degradation by circulating enzymes and prolongs the circulation half-life (Thomas et al., 2020; Zhang et al., 2022). Second, NPs can reduce the toxicity of inclusion by promoting the accumulation of specific sites and reducing untargeted effects. They can be designed to degrade and release their inclusion in the acidic microenvironment of tumors (Whitehead et al., 2014). Finally, NPs can be modified by targeting ligands and other molecules to promote cellular and nuclear uptake and biological distribution to targeted tissues that overexpress targeted proteins (Large et al., 2019). Thus far, a variety of nanomedicine vectors, led by lipid nanoparticles, achieved the growth inhibition of a variety of tumor cells *in vitro* and *in vivo* experiments (Wu et al., 2017; Wu et al., 2018; Mao et al., 2019; Cong et al., 2020; Sun et al., 2020). These vectors can also effectively transfer gene editing tools and carry out gene editing efficiently (Figure 1).

## Applications and Effects of Gene Editing Based on Nano-Drug Delivery Vectors in the Study of Various Malignant Tumors

### Melanoma

Melanoma is the most deadly skin cancer, and the 5-years survival rate of patients with stage IV melanoma is below 15% (Fleming et al., 2015). The incidence of invasive melanoma is still growing faster than any other cancer (Prado et al., 2019), accounting for about 70% of skin-cancer-related deaths (Leonardi et al., 2018). The main cause of death from melanoma is tumor metastasis (Fidler, 2015). In recent years, studies found that the incidence, prognosis, and treatment of melanoma are closely related to CKIT, NRAS, BRAF, and other gene mutations (Nassar and Tan, 2020; Pham et al., 2020). Patients with gene mutation have a poor prognosis, are prone to relapse, metastasis, and other malignant events (Rastrelli et al., 2014; Yde et al., 2018).

**FIGURE 2:**
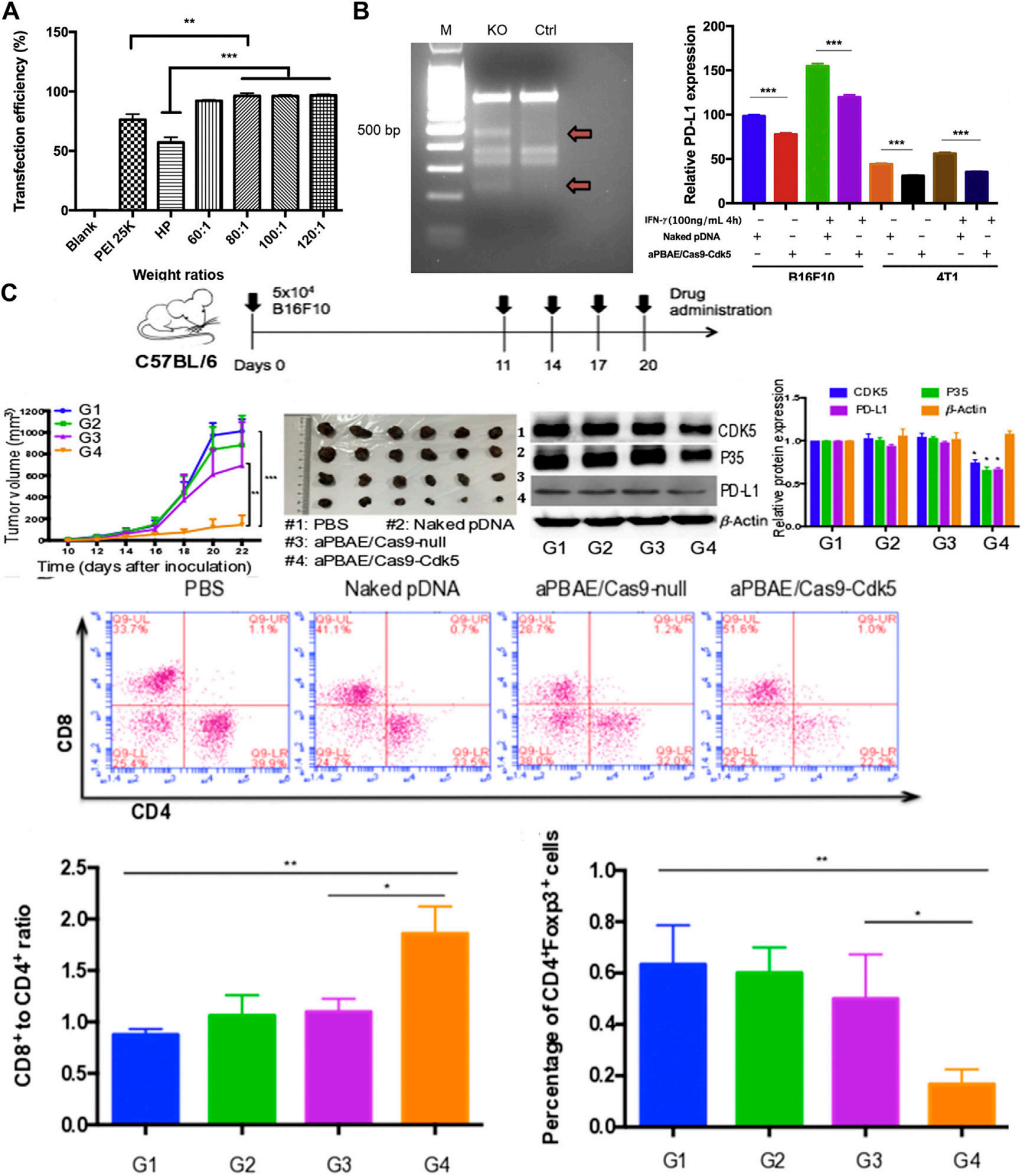
Experiments with aPBAE/Cas9-CDK5 nanocomposites. **(A)** Quantitative analysis of transfection efficiency in B16F10 cells. **(B)** Nanocomposites-mediated cleavage of Cdk5 gene in B16F10 cells detected by T7EI cleavage assay. **(C)** Nanocomposites-mediated PD-L1 attenuation suppresses B16F10 tumor growth and triggers T cell-mediated antitumor immune response in the murine melanoma model. Reproduced with permission from Deng et al. (2020).

Because melanoma is a malignant tumor of the skin, the *in-situ* tumor model can be established through a relatively simple subcutaneous seed tumor. Hence, numerous experiments exploited melanoma as the research object. Deng et al. used poly (β-amino ester) copolymer nanoparticles to carry SpCas9/sgRNA plasmids targeting CDK5 to achieve NHEJ-mediated destruction (Deng et al., 2020). Compared with the PEI 25K and HP transfection reagent, these showed superior transfection efficiency in B16F10 cells (Figure 2A). By effectively knocking out Cdk5 target gene, PD-L1 expression was down-regulated *in vivo*, the CTL mediated immune response was restored, and the immunosuppressive tumor microenvironment was reversed (Figure 2B). Tumor growth was inhibited in B16F10 tumor-bearing mice (Figure 2C). Compared with the anti-PD-L1 antibody, it is capable of maintaining a relatively long-term therapeutic effect. Zhang et al. developed a double-locked nanoparticle called DLNP that can stably exist in blood circulation or normal tissues and release the CRISPR/Cas13a system in the tumor microenvironment with low pH and high H_2_O_2_ concentration. It promotes cellular internalization of the CRISPR/Cas13a system and activation of gene editing after entry into the tumor tissue (Zhang et al., 2019). Improved gene editing efficiency at tumor sites and reduced side effects caused by unintended activation of CRISPR/Cas13a in normal tissue were observed. By systemic administration, the tumor growth of B16F10 tumor-bearing mice was significantly inhibited, and the survival rate was improved.

Plk1 (Polo-like kinase 1) belongs to the Polo-like kinase family, which is a serine/threonine kinase abundant in eukaryotic cells. Overexpression of Plk1 has been found in numerous tumor tissues and model tumor cells (such as A375 cells), and inhibition of Plk1 expression can lead to apoptosis of tumor cells, providing a good strategy for tumor therapy (Lee et al., 2015; Gutteridge et al., 2016; Iliaki et al., 2021). Wang et al. prepared a Cas9 protein/sgPlk1 plasmid carrier with gold nanoclusters as the core and achieved 26.2% Plk1 genome modification *in vitro*, which is more than 10 times more effective than the traditional plasmid transfection method (Wang et al., 2017). The *in vivo* antitumor effect of granulosa was evaluated on the A375 nude mouse subcutaneous tumor model. Compared with other control groups, the nanoparticles had lower toxicity and the most significant inhibitory effect on tumor growth.

**FIGURE 3:**
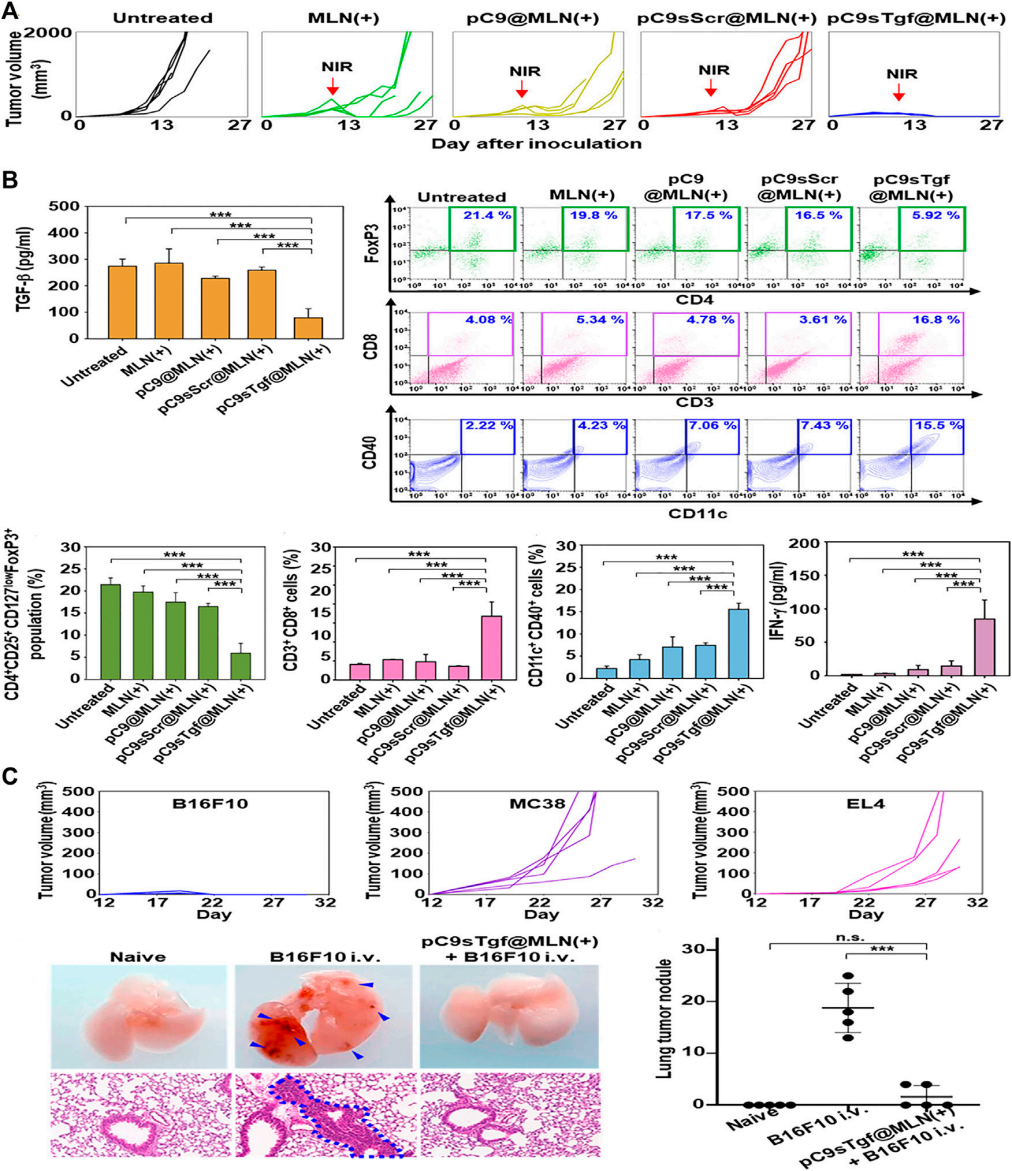
Related experiments of MLN. **(A)** Tumor size changes in each group after unilateral tumor inoculation and treatment. **(B)** Tumor immune microenvironment in each group after unilateral tumor inoculation and treatment. **(C)** Antitumor efficacy against B16F10, MC38, and EL4 distant tumors and B16F10 lung metastasis. Reproduced with permission from Kim et al. (2021).

Photothermal therapy is a treatment method using materials with high photothermal conversion efficiency, implanting them inside the body, gathering them near the tumor tissue with targeted recognition technology, and converting the light energy into heat energy under the irradiation trigger of an external light source (usually near infrared light) to kill cancer cells (Melamed et al., 2015). The advantage of this therapy over traditional cancer therapies is that effective treatments can be performed with precision and few side effects. Numerous studies have addressed this method’s tumor inhibition through photothermal therapy and the interaction of nanoparticles (Hirsch et al., 2003; Dickerson et al., 2008; Chen et al., 2010; Day et al., 2011; Day et al., 2012). Kim et al. constructed a metal-lipid hybrid nanoparticle (MLN) to deliver plasmid DNA of sgRNA and Cas9 proteins encoding TGF-β (Kim et al., 2021). In B16F10 tumor-bearing mice, ifn -γ, cytotoxic T-cells, and mature dendritic cells were increased in the tumor microenvironment by intratumoral injection of the nano-complex plus Near Infra-Red (NIR) irradiation (Figure 3B), which could ablate the primary tumor (Figure 3A) and prevent distant growth of secondary B16F10 cells and lung metastasis (Figure 3C).

### Breast Cancer

**FIGURE 4:**
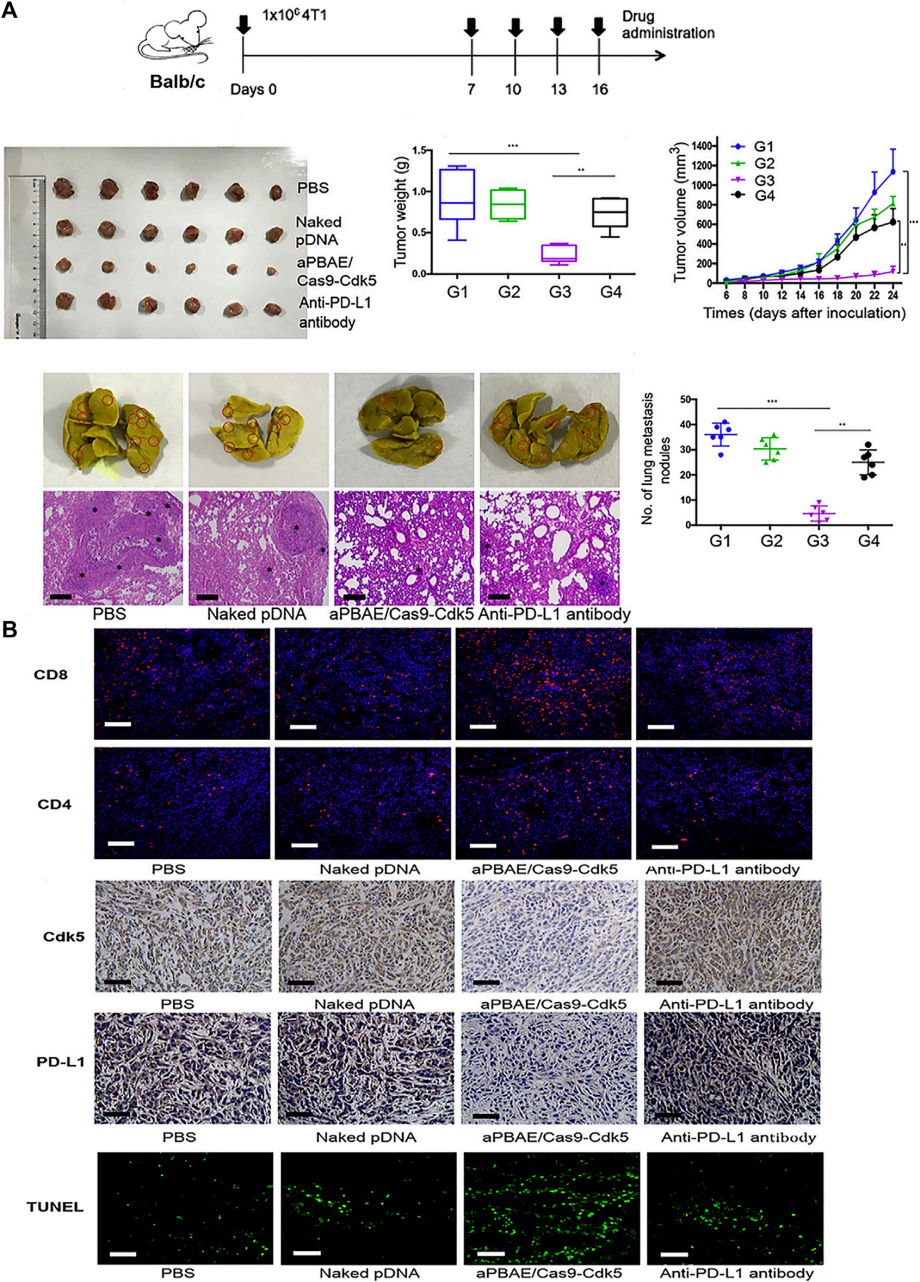
Application of aPBAE/Cas9-CDK5 nanoparticles in 4T1 tumor model. **(A)** Nanoparticles-mediated attenuation of PD-L1 inhibits 4T1 tumor growth and lung metastasis. **(B)** Immunofluorescence, immunohistochemistry, and TUNEL staining of tumor sections. Reproduced with permission from Deng et al. (2020).

Breast cancer is the leading cause of cancer in women, which mostly occurs over 40 years old (Radecka and Litwiniuk, 2016). The incidence of breast cancer is related to genetic and environmental factors (Peairs et al., 2017; Barzaman et al., 2020). According to histological characteristics, breast cancer can be divided into human epidermal growth factor receptor two overexpression (HER2+), hormone receptor positive and three negative breast cancer (TNBC) (Nagini, 2017). Among breast cancers, TNBC is a special breast cancer subtype characterized by deletion of the estrogen receptor, progesterone receptor, and human epidermal growth factor receptor 2 (Foulkes et al., 2010). TNBC lacks effective targeted therapy (Cardoso et al., 2018), is highly invasive and metastatic, and has the highest mortality rate among all breast cancer subtypes (Dent et al., 2007; Foulkes et al., 2010). In recent years, nanomaterials combined with gene therapy has gradually become the focus of TNBC treatment, Chen et al. designed an intelligent nanocomposite that can release CCL25 protein and CD47 siRNA in the tumor tissue of TNBC mouse model. They proved that the transmission of ccl25 in tumor can promote the tumor infiltration of CCR9 + CD8 + T cells by blocking CD47/Sirpα and PD-1/PD-L1 signaling pathways, so as to significantly inhibit tumor growth (Chen et al., 2020). Guo et al. reported a nanolipogel with tumor-targeting, deformable, and non-cationic characteristics, called tNLG, which was used to edit the CRISPR genome in TNBC tumors and successfully inhibited the expression of breast cancer gene Lcn2, mediating a gene editing efficiency of more than 81% (Guo et al., 2019). Deletion of the Lcn2 gene significantly inhibited the migration and mesenchymal phenotype of TNBC cells, thus attenuating the aggressive spread of TNBC. In the TNBC *in situ* tumor model, administration of MDA-MB-231 tumor-bearing mice through the tail vein inhibited 77% of TNBC tumor growth with minimal systemic toxicity. Deng et al. (Fidler, 2015) reported that poly (β -amino esterification) copolymer nanoparticles coated with CDK5-targeting SpCas9/sgRNA plasmids showed superior transfection efficiency in 4T1 cells. (Deng et al., 2020). Compared with the anti-PD-L1 antibody group, pNDA group and PBS group, the tumor volume and weight were significantly reduced in the TNBC lung metastasis model, and the occurrence of lung metastasis was decreased in the nanoparticle group (Figure 4A). Immunohistochemical results showed that the infiltration of CD8^+^ T-cells was high, and the expression of Cdk5 was decreased. TUNEL staining also showed enhanced apoptosis of tumor cells after nanoparticle treatment (Figure 4B).

### Lung Cancer

Lung cancer is one of the most threatening malignant tumors threatening human health and survival (Mao et al., 2016; de Sousa and Carvalho, 2018). In the recent 50 years, the incidence and mortality of lung cancer have been reported in numerous countries, and it has been found to rank in the top two cancer types for both men and women (Romaszko and Doboszyńska, 2018). Surgical treatment is suitable for early stage of lung cancer (Hoy et al., 2019). A study on lung cancer patients showed that tumor suppressor gene mutations and the overexpression of oncogenes may be related to the occurrence of lung cancer (Liu et al., 2004). In recent years, gene editing techniques have been used for *in vitro* experiment of cancer (Jubair and McMillan, 2017). He et al. constructed a natural nanopolymer by combining hyaluronic acid (AHA) functionalization with aptamer (AS1411) and hyaluronic acid (PHA) functionalization with peptide (TAT-NLS) to deliver CRISPR-Cas9 plasmids specifically to tumor cells and achieve effective gene editing. The CTNNB1 gene encoding β -catenin was knocked out, and PD-L1 expression was down-regulated in tumor cells, successfully reversing tumor immunosuppression and immune escape of H1299 (He et al., 2020). Gene-edited tumor cells effectively enhance T cell immunity, including proliferation, cytokine release, and cytolysis activity.

Photothermal therapy (PTT) has attracted increasing attention in the field of tumor therapy owing to its spatio-temporal controlled mode and non-invasive nature (Song et al., 2018). Pan et al. designed a nanocomposite. CRISPR-Cas9 was covalent anchoring of photodegradable 4- (hydroxymethyl) -3-nitrobenzoic acid (ONA) molecules on lanthanum-doped conversion nanoparticles (UCNP), and then coated with polyethylene imine (PEI) to assist the escape of endosomes (Pan et al., 2019). The nano-complex can be effectively internalized by cells *via* the endocytosis pathway, followed by endosomal escape and cytoplasmic release of ucNPS-Cas9 loaded in the cytoplasm. When exposed to NIR, UCNP emit local ultraviolet light and trigger the rupture of the junction. Consequently, Cas9 can be released from the surface of the UCNP and thus enter the nucleus for gene editing. Tumor growth was successfully inhibited in xenografted nude mouse models of A549 cells by targeting oncogene markers (PLK-1 gene). Although PTT can increase the local temperature of the body to over 50°C and kill tumor cells, it can also cause damage to normal tissues and carry the risk of recurrence or metastasis (Gao et al., 2019). Li et al. applied a low oxygen responsive gold nanorods that can carry sgRNA targeting HSP90α (Li et al., 2021). Due to the hypoxic state of the tumor microenvironment, the azo group of the nano-complex is selectively reduced by overexpression reductase, resulting in the release of Cas9 and subsequent HSP90α gene knockout, which reduces the thermal resistance of cancer cells. Under mild PTT conditions, the nano-complex can achieve efficient tumor ablation *in vivo* and *in vitro*. This also reduces thermal damage to normal surrounding tissues.

### Liver Cancer

Hepatocellular carcinoma (HCC) is the third most common cancer worldwide. Surgical resection and liver transplantation are considered to be the only treatments, but are often limited by low liver function and a shortage of liver donors (Waghray et al., 2015; Valverde-López et al., 2018). The epidermal growth factor receptor (EGFR) is a transmembrane receptor that plays an important role in various tumors, especially liver cancer, leading to the growth and proliferation of tumor cells. Sorafenib, a chemotherapy drug, assumes an anti-tumor role by specifically inhibiting multiple molecular targets, such as EGFR and VEGFR2 (Redd Bowman et al., 2020). Zhang et al. designed a polyaminoamine aptamer coated hollow mesoporous silica nanoparticle, which has good stability in blood circulation and a large drug load, and can jointly deliver the Sora and CRISPR/Cas9 system (Zhang et al., 2020). Apt-modified NPs surfaces can effectively enhance the uptake of NPs by HCC cells, thus reducing the side effects of Sora. Moreover, the CRISPR/Cas9 system co-delivered with Sora synergistically inhibits the expression of EGFR and downstream PIK3 Akt pathways, with no detectable off-target effects and a powerful anti-angiogenesis effect. The synergistic efficacy of nanocomposites was studied both *in vitro* and *in vivo*. Both model systems demonstrated that NP has strong cytotoxicity to HCC cells by specifically binding EpCAM receptors on tumor cell membranes. *In vivo* fluorescence imaging shows the accumulation of NPs in the tumor area, while HE staining and blood biochemical analysis showed no significant damage to major organs. Furthermore, effective gene editing of EGFR *in vivo* was confirmed by sequencing, and inhibition of EGFR expression in tumor tissues was detected by IHC.

The PLK1 gene has also been targeted in related studies of liver cancer. Li et al. constructed a proprietary ionizable lipid nanoparticle named iLP181 (Li et al., 2022). Four plasmids containing PLK1-targeting Cas9 protein and sgRNA were designed. PsgPLK1 with the best activity was selected and loaded with iLP181. Studies have shown that iLP181/psgPLK1 is effectively internalized by hepatocellular carcinoma cells by binding ApoE, resulting in long-term *in vivo* and *in vitro* gene editing and significantly inhibiting tumor growth in HepG2-LuC-bearing mice. Compared with Lipo2000 on the market, iLP181 shows strong endogenous escape when delivering nucleic acid.

### Glioblastoma

**FIGURE 5:**
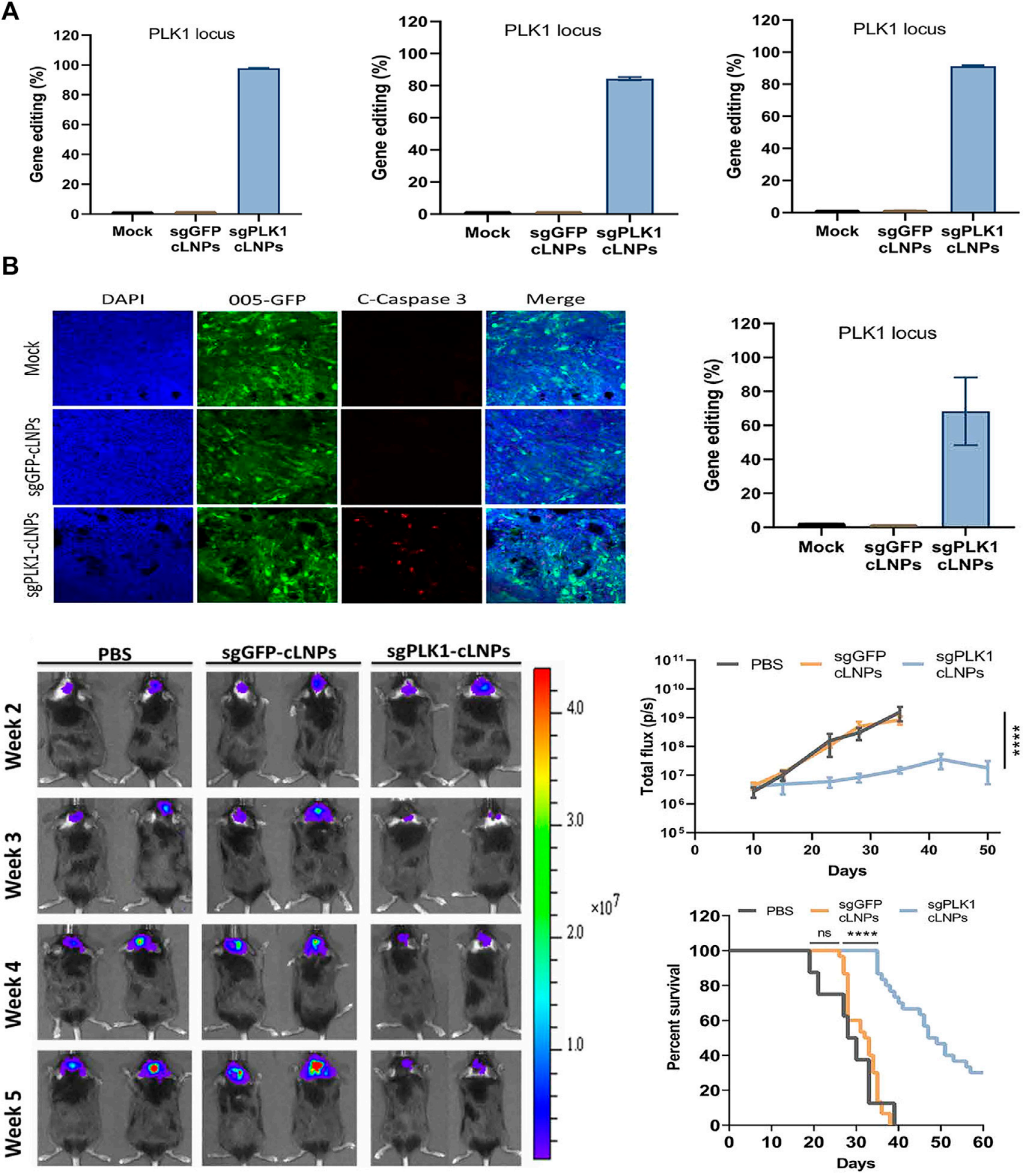
Transfection efficiency of PLK1-targeted CRISPR LNPs (cLNPs) and its application in mouse GBM model. **(A)** Therapeutic genome editing in HEK 293 cells, 005 (murine GBM) and OV8 (human ovarian carcinoma) cells *in vitro*. **(B)** Therapeutic genome editing in 005 GBM bearing mice. Reproduced with permission from Rosenblum et al. (2020).

Glioblastoma (GBM) is the most common and fatal primary brain tumor in adults. The mean survival was only 12–14 months, even after a combination of treatments including surgery, chemotherapy and/or radiation (Davis, 2016). In primary glioblastoma, the molecular changes are mainly the expansion and overexpression of EGFR (Eskilsson et al., 2018), while in secondary glioblastoma, the molecular changes are mainly p53 mutation (Wirsching et al., 2016). BBB block more than 98% of substances from entering the central nervous system and also limit the passage of drugs, including therapeutic drugs. The application of nanocarriers increases the possibility of drugs, nucleic acids, etc. crossing the BBB and further treatment. Yang et al. designed a lipid polymer hybrid nanoparticle (LphNS-CRGD) for efficient and targeted delivery of CRISPR/Cas9 plasmids targeting temozolomide (TMZ) resistance gene O6-methylguanine DNA methyltransferase (MGMT) (Yang et al., 2021). LPHNS-cRGD can target GBM cells and mediate the transfection of pCas9/MGMT to down-regulate the expression of MGMT, resulting in increased sensitivity of GBM cells to TMZ. *In vivo*, local FUS irradiation can safely increase BBB permeability and allow nanoparticles to accumulate in tumors of *in situ* tumor-bearing mice, enhancing the therapeutic effect of TMZ on glioblastoma, inhibiting tumor growth, and prolongating survival of tumor-bearing mice with high biosafety. Co-delivery of Cas9 mRNA and sgRNA using delivery system is a promising strategy to efficiently edit the genome in cells. Kataoka et al. developed a PEGylated polyplex micelle (PM) to co-encapsulate the Cas9 mRNA and sgRNA for genome editing, which could prevent the sgRNA release upon dilution and enhance the tolerability of cas9 mRNA and sgRNA against enzymatic degradation (Uchida and Kataoka, 2019). They further achieved effective genome editing in the mouse brain parenchyma *in vivo* using this PM with co-encapsulated Cas9 mRNA and sgRNA (Abbasi et al., 2021). Rosenblum et al. reported a liposome nanoparticle equipped with PLK1-targeted Cas9 mRNA and sgRNA gene-editing tools, and the gene-editing rate of this system was up to 98% *in vitro* in a variety of cancer cell types (Figure 5A) (Rosenblum et al., 2020). single-dose administration of nanoparticles on tumors in a mouse GBM model resulted in approximately 70% PLK1 gene editing. The induction of apoptosis *in vivo* was assessed by activated Caspase three staining, and increased median survival by approximately 50% and overall survival by 30% in GBM tumor-bearing mice (Figure 5B). Liu et al. synthesized a nanoparticle carrying sgRNAs (Liu et al., 2019). PLys and Cas9/sgRNA complexes make up the core, as well as mPEG, and the core and shell are connected by 2,5-dihydro-2,5-dioxofuran-3-acetic acid (CA). Due to the degradation of CA in acidic environment, the complex exhibits an acid response. In the acidic tumor microenvironment, the mPEG shell can be peeled off, and the inclusion can be efficiently accumulated in the tumor, resulting in gene editing. Tumor growth can be inhibited in heterogeneous tumor models with two cell subpopulations by carrying sgRNAs targeting STAT3 and RUNX1.

### Cervical Cancer

Every year, there are more than 500,000 new cases of cervical cancer worldwide. Infection with human papillomavirus (HPV) causes 90% of cervical cancer cases (Hu and Ma, 2018; Okunade, 2020). HPV is a spherical DNA virus that causes proliferation of the squamous epithelium, the mucous membrane of the human skin. More than 130 species of HPV have been isolated so far. Among them, HPV-16 and HPV-18 are the main drivers of malignant transformation of cervical epithelial cells. To date, treatment outcomes for cervical cancer remain unsatisfactory, with more than 30% of patients initially treated diagnosed with recurrence and metastasis within 2 years and a 5-years survival rate of less than 10% (Pimple and Mishra, 2019). Therefore, it is urgent to develop new strategies to improve the therapeutic treatments of cervical cancer. Zhen et al. developed a novel liposome nanoparticle containing CRISPR/Cas9 gene-editing tool, which can inhibit the proliferation of HPV16-positive cervical cancer SiHa cells and induce apoptosis by inactivating the hr-HPV16E/E7 oncogene (Zhen et al., 2020). Injection of cationic liposomes targeting HPV16 E6/E7 into subcutaneous tumor models in nude mice significantly inhibited tumor growth without significant toxicity.

Gold nanoclusters have good biocompatibility, chemical inertia, strong fluorescence emission, and tunable surface functionalization, which can efficiently complete the delivery task, and are widely used in tumor and other disease models (Vankayala et al., 2015). Tao et al. designed a nanocarrier composed of protamine with AuNC, a gold nanocluster with high biocompatibility, for transporting Cas9 — sgRNA plasmids for genome editing (Tao et al., 2021). The nanocomplex can effectively knock out the oncogenic E7 gene and inhibit the proliferation of HeLa cancer cells. It also can effectively achieve genome destruction in different cancer cells, which significantly broadens its further applications in cancer treatment. In addition, protamine-AuNCs have excellent fluorescence properties, indicating great potential of our nanocarriers for imaging tracking. Ju et al. also proved that AuNCs could be assembled with purified *Streptococcus pyogenes* Cas9 (SpCas9) protein under physiological conditions (Ju et al., 2019). The complex is stable at higher pH and decomposes at lower pH. Due to the low pH microenvironment of the tumor, the assembly-decomposition process can promote the entry of SpCas9 into the tumor nucleus and perform its cleavage function. SpCas9—AuNCs combined with HPV18 E6 sgRNA—effectively knocks out the oncogenic E6 gene, triggering the expression of tumor suppressor protein p53, restoring its function, and inducing the apoptosis of cervical cancer cells. Importantly, the process had little effect on other human cells without the HPV E6 gene, demonstrating the high efficiency and specificity of gene therapy for cancer.

### Ovarian Cancer

**FIGURE 6:**
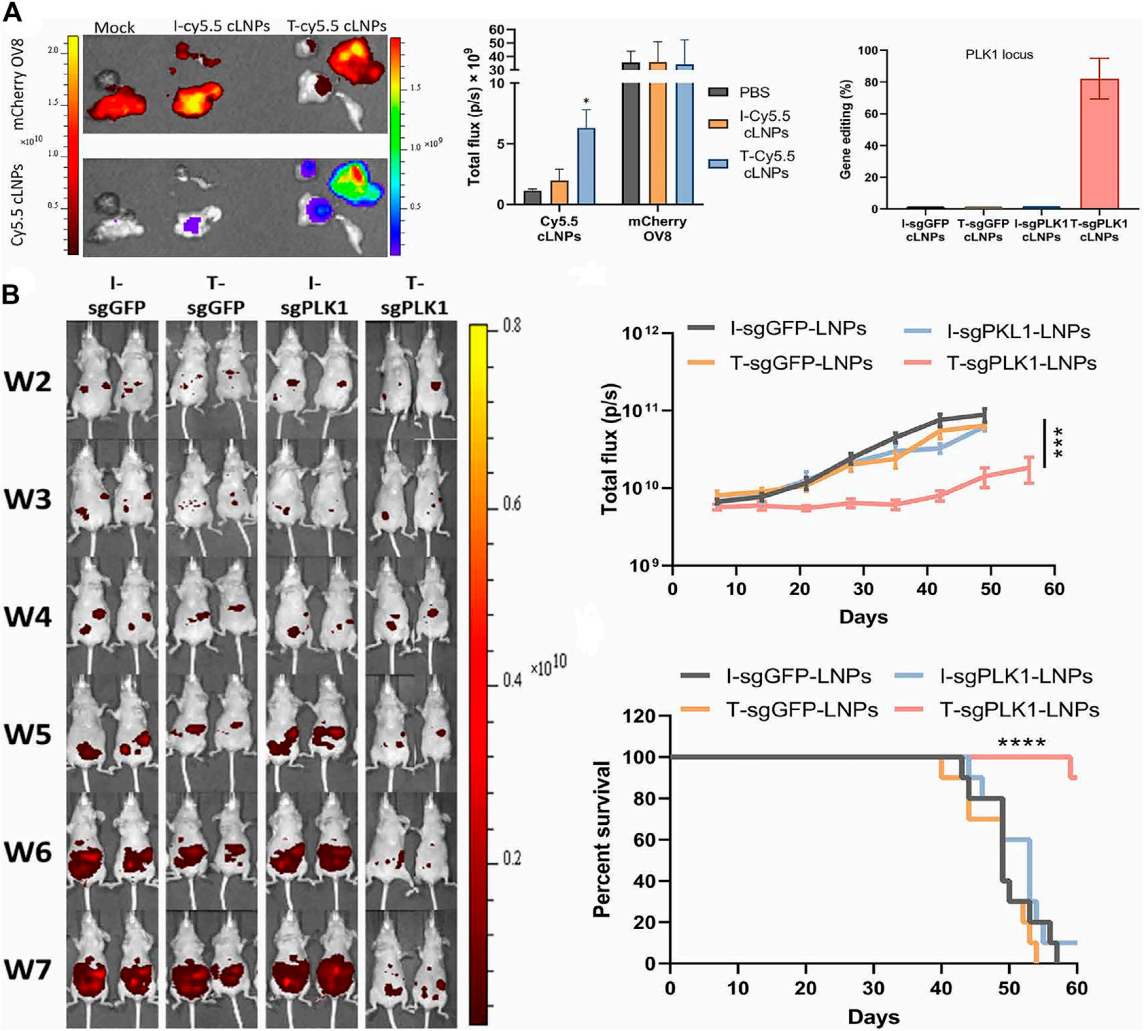
Treatment of Plk1-targeted cLNPs in an OV8-Mcherry mouse model with peritoneal disseminated ovarian cancer. **(A)** Tumor targeting and accumulation of cy5.5-CLNPs in OV8 Tumor-bearing mice. **(B)** Percentage of gene-editing Events in the LOCUS as determined by NGS analysis of PLK1. **(C)** *in vivo* imaging images of OV8-bearing mice. **(D)** Changes in tumor volume. **(E)** Survival curve of tumor-bearing mice. Reproduced with permission from Rosenblum et al. (2020).

Globally, ovarian cancer is the seventh most common cancer in women, with a 5-year survival rate of less than 45%. About 140,000 women die of ovarian cancer every year (Webb and Jordan, 2017; Penny, 2020). Because ovarian cancer lacks specific symptoms in its early stages, and tests do not always yield positive results, most ovarian cancer is not diagnosed until later in life, and many patients have developed malignant peritoneal effusion (Roett and Evans, 2009; Aleksandra Kujawa and Lisowska, 2015). Rosenblum et al. reported a lipid nanoparticle loaded with PLK1 targeted Cas9 mRNA and sgRNA gene-editing tools (Rosenblum et al., 2020). They created a mouse model of peritoneal disseminated ovarian cancer, OV8-Mcherry, and injected nanoparticles through the abdominal cavity. The results showed that the nanoparticles could be selectively ingested into disseminated ovarian tumors (Figures 6A,C), and the gene editing rate was up to 80% *in vivo* (Figure 6B), and the tumor growth was inhibited (Figure 6D), and the survival rate of tumor bearing mice was increased by about 80% (Figure 6E).

### Squamous Cell Carcinoma of the Head and Neck

Head and neck squamous cell carcinoma (HNSCC) is a heterogeneous group of malignant tumors, which is usually caused by drinking and smoking, includes oropharyngeal squamous cell carcinoma (OPSCC), laryngeal squamous cell carcinoma, and oral squamous cell carcinoma (Johnson et al., 2020; von Witzleben et al., 2020). Surgical treatment can lead to visible and functional facial deformities: radiation and chemotherapy are less specific for individual tumors, and have systemic toxic side effects (Murphy et al., 2007). Local or regional metastasis occurs in some patients, which reduces the survival rate to less than 1 year (Schwartz et al., 2000; Forastiere et al., 2001). HuR is an RNA-binding protein encoded by ELAVL1 gene, which plays an important role in regulating the survival, metastasis and drug resistance of HNSCC. Wang et al. designed two different liposome nanocarriers, namely SLN-HPR and LIP-HPR, to deliver CRISPR/Cas9 system and epubicin (Epi), respectively (Wang et al., 2021). These nanoparticles have numerous effects: 1) promoting endosome escape and nuclear localization through the pH response and nucleo-targeted H and R polypeptide sequences; 2) maintaining blood circulation through PEG modified nanoparticles and passively targeting tumors through enhanced permeability and retention effect (EPR); 3) EGFR targeting and cell internalization can be improved by endocytosis mediated by EGFR ligand P; 4) nuclear and/or cytoplasmic release of Epi and/or CRISPR/CAS-9 as a topoisomerase inhibitor HuR knockout system inhibits tumor progression and multiple survival and metastasis pathways, and regulates drug resistance. By establishing a SAS/LUC-BEARING mouse model, HuR CRISPR/SLN-HPR pre-knocked out HuR in SAS cells and co-treated SAS/LUC mice with Epi/LIP-HPR, achieving the most significant antitumor effect.

## Summary

Nano-drug delivery vectors can efficiently deliver gene-editing tools for a variety of malignancies, and inhibit tumor cell growth *in vitro* and *in vivo* experiments. Although most of results have been encouraging, the gene therapy based on nano-drug delivery vectors is currently only confirmed in animal trials, and no clinical trials have been conducted to date. Numerous problems and conditions must be solved and perfected before clinical treatment can be realized. First of all, in most of *in vivo* experiments, there are few *in situ* models, but most of them are subcutaneous models. It has little effect on melanoma, breast cancer and other visible tumors, but this is not the real growth pattern of more tumors. We still need better modeling methods and tumor observation methods, and pay attention to the humanization of animal models in order to simulate more realistic tumor growth in humans (Hu et al., 2016; De La Rochere et al., 2018; Ito et al., 2018; Tian et al., 2020). Secondly, in terms of inclusion, although CRISPR/Cas9-based gene-editing technology is stable, efficient, simple, and widely used, some problems remain, such as the off-target effect, targeted mutation, and immune response of human body to bacteria-derived Cas9 protein (Zhang H. et al., 2021). One of the most promising applications of CRISPR/Cas9 in gene therapy is CAR T-cell therapy. However, clinical studies have found that this treatment is neurotoxic *in vivo* and may lead to cytokine release syndrome (Zhang M. et al., 2021). Nanoparticles as carriers likewise must address some challenges. First, NPs carrying gene-editing tools must successfully edit a sufficient number of cells to achieve the desired therapeutic outcome, which can be met by linking the targeted ligand to the NPs to facilitate its binding and uptake to the targeted cells (Schmid et al., 2017). Second, we need NPs to accurately control the delivery and release time of gene-editing tools (Yin et al., 2016). This challenge can be met by developing NPs using materials with highly adjustable degradation curves to release the inclusion on demand (Kamaly et al., 2016). Finally, materials used in clinical trials and even clinical treatment must have a high level of safety (Kamali Shahri et al., 2021; Martín-Sabroso et al., 2021; Phillips and Mousa, 2022). In addition, the use of nanoparticles to deliver CRISPR/Cas9 to acute myeloid leukemia (AML) and chronic myeloid leukemia (CML) genes in animal models has been shown to be a promising strategy for the treatment of leukemia (Liu et al., 2018; Ho et al., 2021; Ren et al., 2022). However, there are few reports about non-solid tumors. Despite the difficulties, it is expected that in the near future, animal trials, clinical trials, and even clinical treatments for better tumor modeling will begin to yield exciting results.
